# Downregulation of KLF8 expression by shRNA induces inhibition of cell proliferation in CAL27 human oral cancer cells

**DOI:** 10.4317/medoral.18736

**Published:** 2013-05-31

**Authors:** Zhang Bin, Li Ke-Yi, Zhang Wei-Feng, Jiang Li-Cheng, Liu Xian-Bin, Xia Chun-Peng, Yuan Dao-Ying, Liu Shu-Wei

**Affiliations:** 1 Department of Anatomy, Shandong University School of Medicine, Jinan 250012, Shandong Province, P. R. China; 2 Liaocheng People’s Hospital and Liaocheng Clinical School of Taishan Medical University (Shandong Province Key Laboratory of Oral and Maxillofacial-Head and Neck Medicine), Liaocheng 252000, Shandong Province, P. R. China

## Abstract

Objectives: KLF8 is a member of KLF transcription factors which play an important tolr in oncogenesis. It is barely expressed in normal human epithelial cells but highly overexpressed in several types of human cancer cell lines. In the present study, we investigate the role of KLF8 in oral cancer and the effects of KLF8 knockdown via lentivirus mediated siRNA infection in human adenosquamos carcinoma CAL 27 cells. Study Design: We developed a vector-based siRNA expression system that can induce RNAi in CAL 27 oral cancer cells. Downregulation of KLF8 was confirmed by evaluating GFP expressions, RT-PCR and western blot analysis. Finally, the effects of KLF8 downregulation were analyzed by MTT assay and colony formation assays. Results: The expression levels of KLF8 mRNA and proteins are reduced in CAL 27 cells that transfected with 21-nt siRNA against KLF8. Lentivirus-mediated silencing of KLF8 reduces cell proliferation and colonies number, thereby indicating the role of KLF8 in cell proliferation and tumorigenesis. Conclusions: These results strongly suggest that KLF8 is essential for growth of CAL 27 cancer cells. A better understanding of KLF8 function and processing may provide novel insights into the clinical therapy of oral cancer.

** Key words:**KLF8, lentivirus, CAL 27, oral cancer, cell proliferation.

## Introduction

The cancer occurring in the tissue of oral cavity or oropharynx is termed as oral cancer. Oral cancer is the sixth most common cancer worldwide and continuously represents a serious public health problem. It is the most common neoplasm of the head and neck with the annual incidence of new cases exceeds 300,000 worldwide (1). More than 90 percent of these tumors are squamos cell carcinomas, which arise from mucosal lining and characterized by the simultaneous presence of glandular and squamos component (2,3). All cases of oral cancer have shown an aggressive course with 60% of patients dying of disease.

Surgical treatment is the mainstay of therapy for oral cancer patients, particularly in advanced stages of cancer. External beam radiation therapy and brachytherapy have been used as the primary modality for treating patients with early stage oral cancer (4). Despite therapeutic advances, including surgery, radiation, and chemotherapy, the five-year survival rate for oral cancer has not improved significantly in the last three decades (5). This disappointing outcome strongly suggests that novel targeted therapeutic agents are urgently needed to improve the treatment of patients diagnosed with oral cancer.

Recent studies have clearly demonstrated advantages of RNA interference (RNAi) for the growth suppression and killing of cancer cells (6). RNAi is a phenomenon leading to post-transcriptional gene silencing (PTGS) after endogenous production or artificial introduction into a cell of small interfering double stranded RNA (siRNA) with sequences complementary to the genes. A number of gene products involved in carcinogenesis have already been explored as targets for RNAi, and RNAi targeting of molecules crucial for tumor-host interactions and tumor resistance to chemo- or radiotherapy has also been investigated. The silencing of gene associated with cancer by RNAi has generated significant antiproliferative effects in cell-culture systems or in preclinical animal models (7).

Krüppel-like factor 8 (KLF8) is a GT-box (CACCC) binding dual-transcription factor that has critical role in the regulation of the cell cycle progression, transformation and invasion. They belongs to the family Krüpple-like zinc finger transcription factors, which in humans comprises at least 24 members (8). KLF8 has been demonstrated as focal adhesion kinase (FAK) downstream effectors. Similar with FAK, KLF8 is ubiquitously expresses in invasive human cancer. KLF8 plays an important role in oncogenic transformation in several types of human cancer such as breast cancer, gastric cancer (9,10). KLF8 knockdown has been demonstrated to reduce the proliferation and invasion of human gastric cells. However, effects of KLF8 downregulation in human oral cancer invasion have not been investigated. Hence, in this study we investigate the role of KLF8 in oral cancer and the effects of KLF8 knockdown via lentivirus mediated siRNA infection in human adenosquamos carcinoma cells (CAL 27).

## Matherial and Methods

1.1. Cell culture

Human adenosquamous carcinoma cell line CAL 27 was bought from American Tissue Culture Collection (ATCC). Cells were grown in DMEM (Gibco) with 10% fetal bovine serum (FBS; Gibco), 100 μg/ml penicillin, and 100 μg/ml streptomycin in a humidified 37 °C incubator with 5% CO2.

1.2. Design and construction of recombinant lentivirus

siRNA for KLF8 (5’-CAGCACTGTTTAATGACAT-3’) was inserted into the Plasmid pFH-L (Shanghai, Hollybio, China). Non-silencing RNA (5’-TTCTCCGAACGTGTCACGT-3’) was used as control.

1.3. Lentivirus packing and infection

The lentivirus expression plasmid (pFH-L-KLF8 or control vector), together with pVSVG-I and pCMVΔR8.92 plasmids that contained the imperative elements for virus packaging, were co-transfected into 293T cells with lipofectamine 2000, according to the manufacturer’s instructions for the generation of KLF8-shRNA lentivirus or control lentivirus. Lentivirus was harvested at 48 h post-transfection, centrifuged to get rid of cell debris, and then filtered through 0.45 µm cellulose acetate filters followed by ultracentrifugation. For lentivirus infection, CAL 27 cells were grown to 70–80% confluence and infected with KLF8-shRNA lentivirus or control lentivirus at MOI of 10. In order to determine the infection efficiency, cells expressing GFP protein were observed using fluorescence microscopy (CKX41, Olympus) 5 days after infection.

1.4. Colony formation assay

In brief, 0.5 ml underlayers consisting of 0.8% agar medium were prepared in six-well plates. Uninfected and infected cells were trypsinized, centrifuged, resuspended in 0.4% agar medium (equal volumes of 0.8% noble agar and culture medium), and plated onto the top agar at 200 cells per well. The cells were cultured for 14 days at 37°C. After cultured for 14 days, adherent cells were washed twice with PBS, fixed with 4% paraformaldehyde for 30 min at room temperature. Colonies were visualized using a cell staining Giemsa solution (Chemicon) and counted under a fluorescence microscope.

1.5. Cell viability assay

Cell viability was determined by MTT reduction assay. In brief, uninfected and infected cells cells were seeded into 96-well plates at a density of 2 × 104 cells/well and incubated with serum free medium. After infection for 1, 2, 3, 4 and 5 days, 100 µl of MTT (0.5 mg/ml final concentration) was added and incubation was continued for another 4 hours. MTT is used as an indicator of cell viability through its mitochondrial reduction to formazan. Mitochondrial succinate dehydrogenase in live cells converts MTT into visible formazan crystals during incubation. The formazan crystals were then solubilized in dimethylsulphoxide (DMSO) and the absorbance was measured at 595 nm by using enzyme-linked immunosorbent assay (ELISA) microplate reader (Bio-Rad 680, Bio-Rad USA). Each assay was done in triplicate.

1.6. Reverse Transcriptase-Polymerase Chain Reaction (RT-PCR) analysis

Total RNA was extracted from cells by using TRIzol reagent according to the manufacturer’s instruction. Reverse transcription was performed using MMLV Reverse Transcriptase and oligo (dT) primer. RT-PCR was conducted with the TAKARA TP800-Thermal Cycler DiceTM Real-Time System using SYBR green Master Mixture. All amplifications were performed in the final reaction mixture (20 µl). Single stranded cDNA was amplified by PCR with specific primers for KLF8 and GAPDH. Primer sequences used to amplify the containing were: KLF8 sense 5’-GGGTGTTTGGCTTCTTTGC-3’, KLF8 antisense 5’-GGCTGTGGTCTCATCTGC-3’; GAPDH sense: 5’-TGACTTCAACAGCGACACCCA-3’, GAPDH antisense: 5’-CACCCTGTTGCTGTAGCCAAA-3’. The following PCR conditions were applied for all amplifications: 25 cycles of dena-turation at 94 °C for 30 s, annealing at 57 °C for 30 s, and extended at 72 °C for 30 s. The resulting cDNA were separated by electrophoresis on 1% agarose gel for 15 minutes at 100 V, followed visualization under UV light after ethidium bromide staining. Band intensities were quantifies with Multi gauge Software (Fujifilm Life Science, Tokyo, Japan) and band of specific genes were normalized using GAPDH as references.

1.7. Western blot analysis

Standard procedures were used for western blotting, where CAL 27 cells were washed with ice-cold PBS, scrapped and then lysed in RIPA buffer. Cell debris was removed by centrifugation followed by quick freezing of the supernatants. The protein concentrations were determined according to the Bicinchonic acid (BCA) assay. Total protein (30 µg) was subjected to 10 % SDS-PAGE gel electrophoresis and electro-blotted onto PVDF membrane. The membrane were blocked with 5% bovine serum albumin (BSA) for 2 h and the incubated with different antibodies which were used to detect respective proteins using a chemiluminescent ECL assay kit, according to the manufacturer instructions. Western blots were visualized using a LAS3000® Luminescent image analyzer and protein expression was quantified by Multi gauge V3.0 software (Fujifilm Life Science, Tokyo, Japan).

## Results

2.1. Effect of lentivirus mediated si-KLF8 infection on KLF8 expression in CAL 27 cells

**Figure 1:**
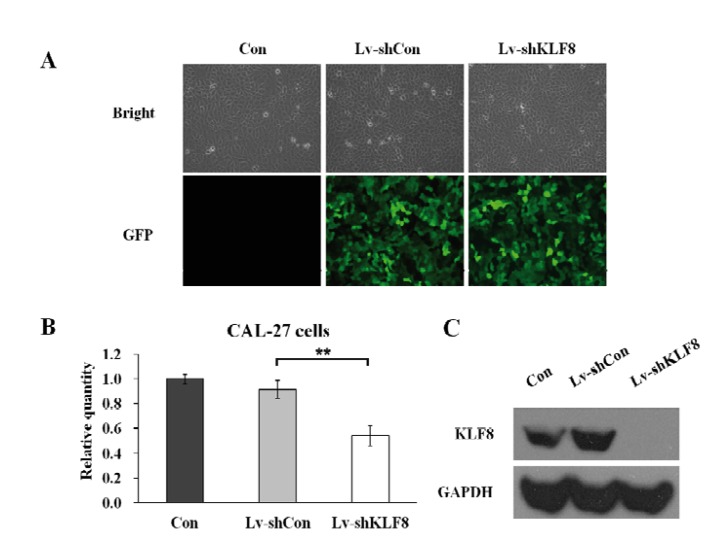
Down regulation of KLF8 in CAL 27 cells by siRNA. Transduction efficiency was assessed four days after infection at MOI of 10; magnification 200×. Light microscope (up); fluorescent microscope (bottom) (A). Total cellular RNA was extracted four days after infection and determined by RT-PCR analysis. Data was expressed as fold changes (B). Total cellular proteins was extracted and determined by western blot (C). GAPDH was used as control. Data represent the means of three independent experiments. Con: uninfected cells; Lv-shCon: control lentivirus infected cells and Lv-shKLF8: KLF8-shRNA lentivirus infected cells.

RNAi targeted gene which regulated cell proliferation could be an important way to prevent oral cancer cell invasion. KLF8 have been shown to play significant roles in the regulation of the cell cycle progression, transformation and invasion. In this study, GFP containing lentivirus mediated infection of KLF8 siRNA in CAL 27 cells was performed. The successful infection was confirmed by evaluating the expression of GFP. As shown in figure 1, the delivery efficiency of control lentivirus infected cells (Lv-shCon) and KLF8-shRNA lentivirus infected cells (Lv-shKLF8) are approximately 85% at multiplicity of infection (MOI) of 10. Most of CAL 27 cells expressed GFP protein when observed under fluorescence microscope 4 days-after infection, which suggest that the infection is successful.

Furthermore, the effects of lentivirus-mediated silencing of KLF8 gene and protein expression were evaluated by RT-PCR and western blot analysis, respectively. The level of GAPDH determined that equal quantities of KLF8 loaded in the gel. Quantitative analysis of KLF8 mRNA level by densitometry revealed a significant decrease in Lv-shKLF8 (0.58-0.6 fold) compared to control and Lv-shCon (Fig. 1). Consistent with mRNA level result, western blot analysis showed that KLF8 protein expression in Lv-shKLF8 groups also downregulated. However, Lv-shCon group expressed KLF8 protein in almost similar level with the control group; suggesting that KLF8 specifically knocked down by KLF8-specific shRNA in CAL 27 cells.

2.2. Effect of lentivirus mediated si-KLF8 infection on CAL 27 cells proliferation

**Figure 2:**
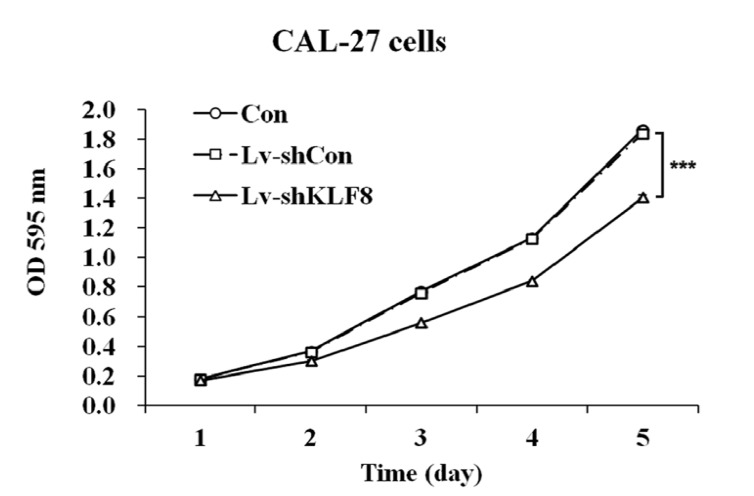
siRNA-mediated downregulation of KLF8 in reduces proliferation of CAL 27 cells. A growth curves of cells during five days evaluated by MTT assay. Data represent the means of three independent experiments. Con: uninfected cells; Lv-shCon: control lentivirus infected cells and Lv-shKLF8: KLF8-shRNA lentivirus infected cells.

In the present study, the standard MTT assay was used to assess the effect of the si-KLF8 on proliferation of CAL 27 cells (Fig. 2). Starting from the second day to fifth day after infection, Lv-shKLF8 infected CAL 27 cells showed a decrease in proliferation relative to that of uninfected cells (con) and controls (Lv-shCon). These results strongly suggest that siRNA-mediated downregulation of KLF8 is specifically and effectively reduces proliferation of CAL 27 cells.

2.3. Effect of lentivirus mediated si-KLF8 infection on colony forming capacity of CAL 27 cells

**Figure 3:**
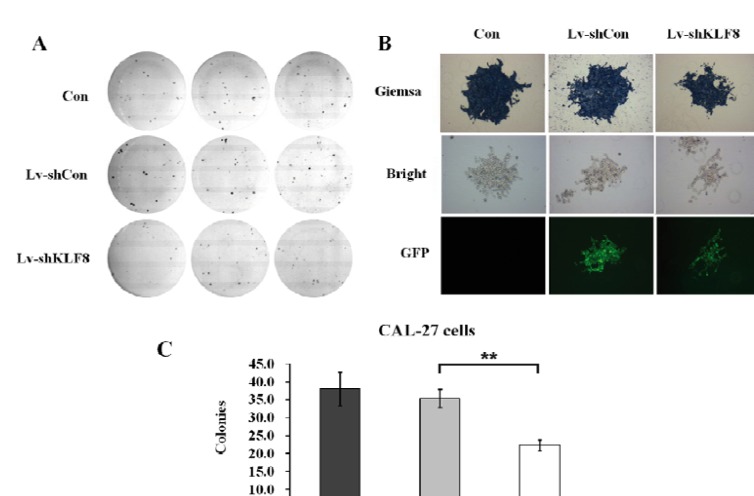
Silencing of KLF8 suppressed the colony-forming capacity of CAL 27 cells. Colony-formation assays of uninfected cells (upper panels); Lv-shCon (lower panel) and Lv-shKLF8 (lowest panels) cells (A). All uninfected cells and infected cells were cultured for 14 days in the presence and the numbers of colonies were counted by Giemsa staining (magnification 200×). (B). Quantitative analysis of colony formation assay (C). Data represent the means of three independent experiments. Con: uninfected cells; Lv-shCon: control lentivirus infected cells and Lv-shKLF8: KLF8-shRNA lentivirus infected cells.

Colony formation is commonly used to assess the proliferative capacity of cancer cells. In several studies, this capacity has been shown to indicate tumorigenic potential. Therefore, colony-forming capacity of uninfected cells, Lv-shCon, and Lv-shKLF8 infected cells were tested. The colony formation assay demonstrated that downregulation of KLF8 suppressed the colony-forming capacity of CAL 27 cells (Fig. 3). Moreover, giemsa staining and fluorescence microscopy (14 days after infection) revealed that stable infection of Lv-shKLF8 resulted in dramatic decrease in the number and size of colonies in CAL 27 cells (Fig. 3). Compared to the control (37 colonies) and Lv-shCon group (35 colonies), the cell number per colony of Lv-shKLF8 group were reduced to 22 colonies (Fig. 3). These results indicate that KLF8 is essential for growth of CAL 27 cancer cells.

## Discussion

In some European and Southeast Asian countries, oral cancer prevails to be the leading form of cancer. Poor survival outcome with a probability of survival at 5 years being less than 50 percent is seen in oral carcinoma diseases. High recurrence rate leading to treatment failure seems to be the major drawback in the patient treatment protocol (11). Therefore novel targeted therapeutic methods such as gene therapies are opening a new way to improve the treatment of patients diagnosed with oral cancer.

KLF8 is a member of KLF transcription factors which play an important role in oncogenesis. KLF8 is barely expressed in normal human epithelial cells but highly overexpressed in several types of cancer cell lines established from human patients, including ovarian, gliomas, breast, gastric and renal carcinomas (12). Various signaling pathways regulating KLF8 expression and its target genes associated with cancer have been identified. For example, KLF8 has been reported to transduce FAK to PI3K to AKT to SP1 signaling to up regulate cyclin D1 expression and enhance the cell cycle progression in ovarian cancer cell (13). Li et al. (16) reported the role of KLF8 in mediating Wnt to β-catenin signaling pathway to activate the transcription of c-Myc, cyclin D1 and Axin1 which promote HCC cell proliferation. In addition, KLF8 is necessary for gastric cancer cell survival and invasion. Furthermore, silencing of KLF8 may lead to cancer cell death (10). KLF8-knockdown by shRNA in U87 glioma cells has been reported to suppressed tumor cell proliferation (14). However, silencing of KLF8 has not been explored in oral cancer cells.

In the present study, we have developed a vector-based siRNA expression system that can induce RNAi in CAL 27 oral cancer cells. We directed 21-nt siRNA against KLF8 in order to simultaneously suppress KLF8 expression. We have shown that the expression levels of KLF8 mRNA and proteins are reduced in transfected cells. Lentivirus-mediated silencing of KLF8 reduces cell proliferation and colonies number, thereby indicating the role of KLF8 in cell proliferation and tumorigenesis. These result, provide the first evidence for the potency of this transcription factor in human oral cancer. In agreement with our finding, several studies demonstrated that knocking down KLF8 in the U87-MG glioma cells, 786-0 renal cells, and SGC-7091 gastric cancer cells, HCC hepatocellular carcinoma dramatically inhibited the cells proliferation (10,14-16). KLF8-siRNA has been reported to depress the cellular growth and invasion of 786-0, U87-MG and SGC-7091 cells in vitro. Flow cytometry results revealed that KLF8-siRNA could induce an increase in G0/G1 phase cells and induce cancer cell apoptosis (10). KLF8 knockdown in the cancer cells resulted in reduced xenografted tumor growth in nude mice with decreased expression of cyclin D1 and Bcl-2, the upregulation of pro-apoptotic gene expression and apoptosis in the tumor cells (17).

In conclusion, the present study demonstrated that the RNAi-mediated targeting of KLF8 suppressed CAL 27 cell proliferation and colony number. These results also strongly suggest that the siRNA-mediated downregulation of target gene expression is sufficiently stable in oral cancer cells. Furthermore, a better understanding of KLF8 function and processing may provide novel insights into the clinical therapy of oral cancer.
